# Posterior Reversible Encephalopathy Syndrome Potentially Associated With Erythropoietin Analogs: A Case Report

**DOI:** 10.7759/cureus.91599

**Published:** 2025-09-04

**Authors:** Ayman Shaat, Avinash K Jha

**Affiliations:** 1 Critical Care Medicine, Royal Preston Hospital, Preston, GBR

**Keywords:** causation, convulsion, dialysis patients, epileptic encephalopathy (ee), erythropoietin, posterior reversible encephalopathy syndrome (pres)

## Abstract

Posterior reversible encephalopathy syndrome (PRES) is a potentially life-threatening but reversible neurological disorder. Commonly seen in the intensive care unit (ICU), PRES can arise from uncontrolled hypertension, renal failure, or the use of immunosuppressants and cytotoxic drugs. Erythropoietin (EPO) analogs help manage anemia in chronic kidney disease, but their hypertensive effects may lead to PRES.

We report a patient with end-stage renal disease presenting with PRES that occurred four months after commencing darbepoetin alfa and closely correlated with darbepoetin use. Brain MRI confirmed the diagnosis. Discontinuing the drug and managing blood pressure (BP) resulted in complete neurological recovery. Re-challenging the patient upon recurrence strengthened the causal link. Therefore, we emphasize that healthcare providers should maintain a high index of suspicion for PRES related to EPO in patients in the ICU with hypertension who develop neurological symptoms.

## Introduction

Posterior reversible encephalopathy syndrome (PRES) is a clinico-radiological condition characterized by the presence of acute neurological signs such as seizures, altered sensorium, headache, and visual disturbances. Radiographically, it manifests as vasogenic edema, primarily involving the posterior cerebral hemispheres [1,2]. PRES is often associated with hypertension, renal failure, and immunosuppressant agents [2,3]. Erythropoietin (EPO) and its derivatives are frequently used to treat anemia in chronic kidney disease. However, blood pressure (BP) may increase, and in rare cases, hypertensive crisis and encephalopathy can develop [4,5]. Earlier case reports include a case series of six patients and another with a single case report mentioning the association of PRES with EPO, making the incidence rare and underrecognized [2,6,7]. The possible mechanisms include EPO-induced alterations in cerebral blood flow, worsening of hypertension, and changes in the integrity of endothelial cells [7]. We present a case of PRES in a patient admitted to the intensive care unit (ICU) with a strong association to darbepoetin administration.

## Case presentation

A man in his 60s presented to the clinic with slurred speech, headache, and hypertensive urgency with a BP of 200/80 mmHg. He was urgently transferred to the emergency department (ED) for further management. The symptoms resolved with antihypertensive therapy, and he was discharged home with his BP in the normal range. His past medical history included hypertension and hyperlipidemia under treatment for the past 20 years. Four months earlier, he was referred to our hospital with bilateral pyelonephritis and end-stage renal disease (ESRD) and was started on long-term hemodialysis. He was started on darbepoetin alfa 50 µg weekly by the subcutaneous route for the first two doses and then transitioned to 50 µg weekly by the intravenous (IV) route.

**Figure 1:**
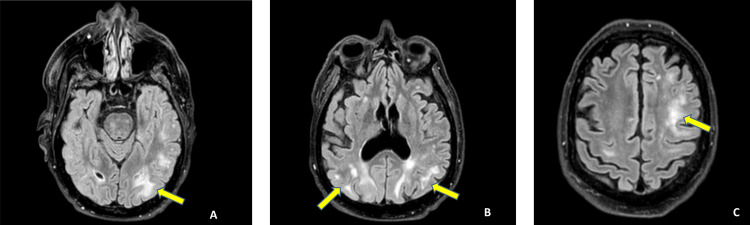
T2-FLAIR MRI of the brain showing features of PRES. Yellow arrows indicate areas of hyperintensity consistent with vasogenic edema in the parietal and occipital lobes. (A and B) PRES-associated changes in the occipital lobe. (C) PRES-associated changes in the parietal lobe. PRES, posterior reversible encephalopathy syndrome; FLAIR, fluid-attenuated inversion recovery

He was readmitted to the ED 48 hours after discharge. On assessment, he had an altered mental status, dysphasia, and new-onset seizures. His airway was secured, and he was mechanically ventilated; subsequently, he was transferred to the ICU for further management. CT brain and CT brain angiography performed were normal. Lumbar puncture results were within normal limits. The patient remained encephalopathic despite being normotensive on a combination of antihypertensive medications. Hence, an MRI of the brain was performed after 12 hours of admission to the ICU. Brain MRI revealed bilateral parieto-occipital T2-FLAIR hyperintensities, consistent with PRES (Figure 1).

He underwent tracheostomy after seven days in the ICU, and the sedation was weaned gradually. He recovered from his symptoms, and after 12 days in the ICU, he was conscious and had no abnormal neurological signs or symptoms.

On ICU day 26, the patient developed agitation, confusion, and hypertension (BP 170/110 mm Hg) three hours after completion of a dialysis session. His agitation was managed with IV antihypertensive and dexmedetomidine infusion. The past medication history revealed that darbepoetin alfa had been transitioned to the IV route with a hemoglobin target of 11 g/dL. On further evaluation, it was evident that the patient had received darbepoetin during dialysis earlier that day. Following this, the EPO analog was discontinued. There were no additional neurologic events.

**Figure 2:**
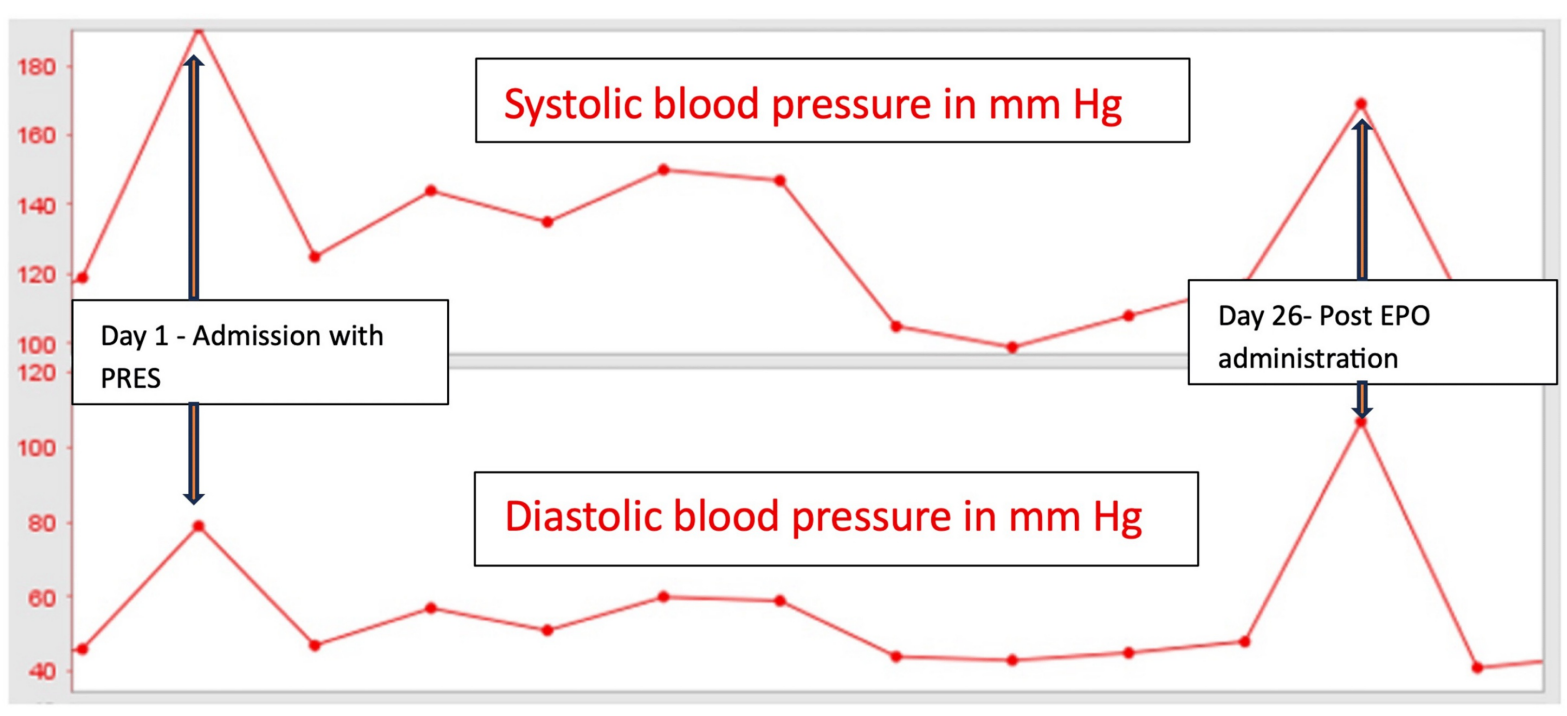
Trend of blood pressure and timing of EPO analog (darbepoetin alfa) administration. EPO, erythropoietin analog (darbepoetin alfa); PRES, posterior reversible encephalopathy syndrome

**Figure 3:**
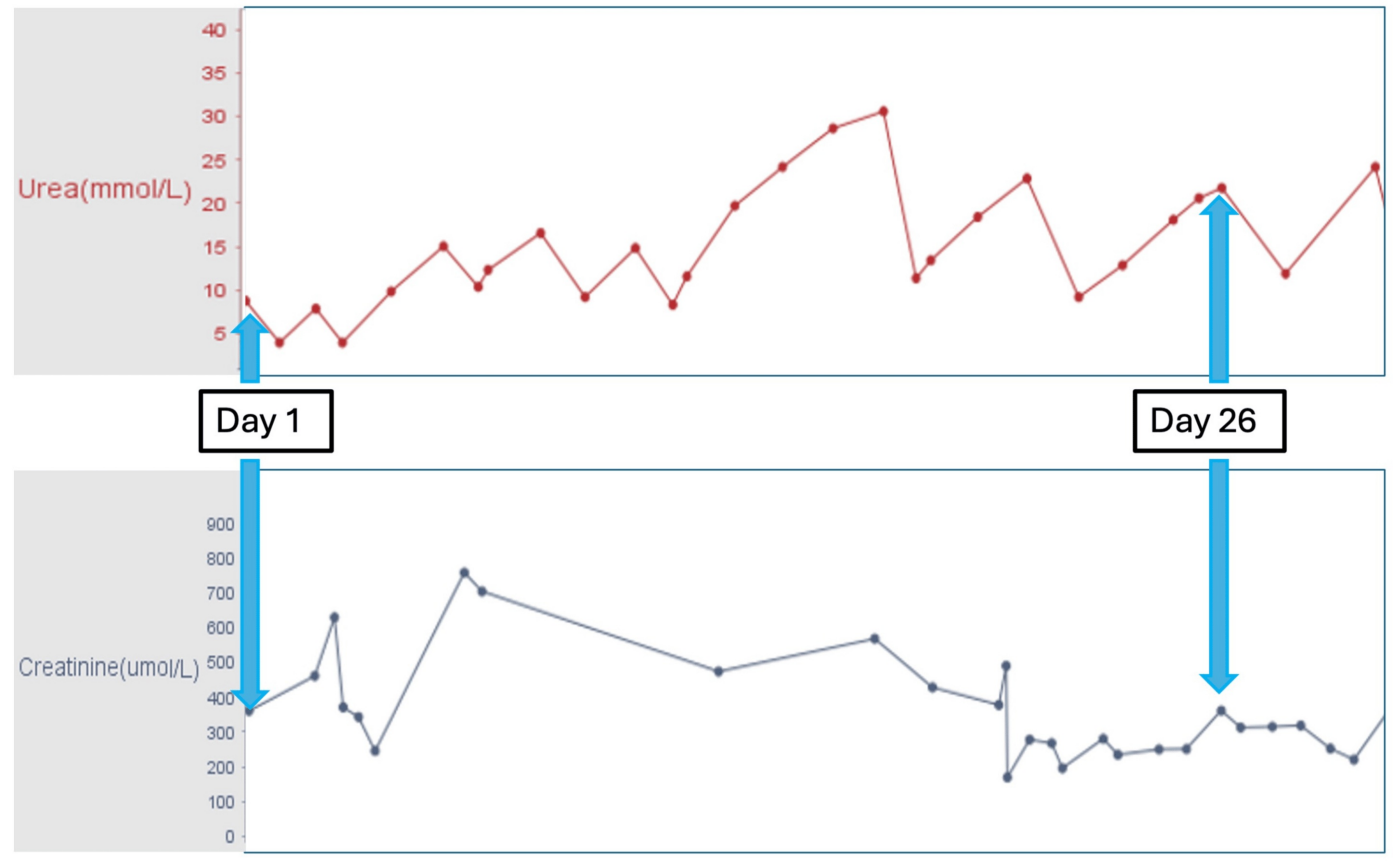
Trends of urea and creatinine during the intensive care unit admission. Day 1: Admission to the intensive care unit with symptoms of posterior reversible encephalopathy syndrome (PRES). Day 26: Recurrence of PRES symptoms following administration of darbepoetin alfa.

Etiologies such as autoimmune conditions, dialysis disequilibrium syndrome, and sepsis were ruled out as differential diagnoses. Additionally, delirium was excluded due to the brain MRI findings and the temporal relationship between the symptoms and the re-administration of darbepoetin alfa. Following decannulation of the tracheostomy and ICU discharge on day 31, the renal team assumed care, and consideration was given to reintroducing darbepoetin alfa at lower doses via subcutaneous administration. A subsequent MRI was scheduled for three months later. The patient remained neurologically intact. The trends in the patient’s blood pressure, and timing of EPO analog administration are shown in Figure 2. The trends of urea and creatinine are shown in Figure 3.

## Discussion

PRES results from cerebral vasogenic edema due to failure of autoregulation, typically arising from acute severe hypertension. The pathophysiology includes factors such as endothelial dysfunction, vasospasm, and disruption of the blood-brain barrier [2,3].

**Figure 4:**
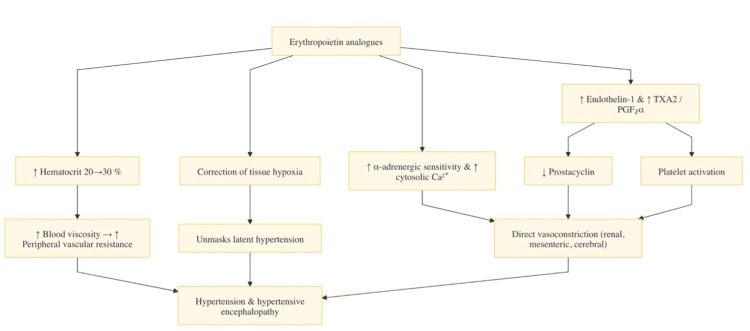
Multifactorial mechanisms of PRES caused by EPO analogs. Image credit: Avinash K. Jha. TXA2, thromboxane A2; PGF2a, prostaglandin F 2alfa; Ca2+, calcium; EPO, erythropoietin; PRES, posterior reversible encephalopathy syndrome

EPO analogs increase systemic vascular resistance through various mechanisms, including the suppression of nitric oxide (NO), elevated plasma endothelin-1 levels, and increased blood viscosity [4,6,7]. Figure 4 illustrates the various mechanisms by which EPO analogs can cause PRES.

**Figure 5:**
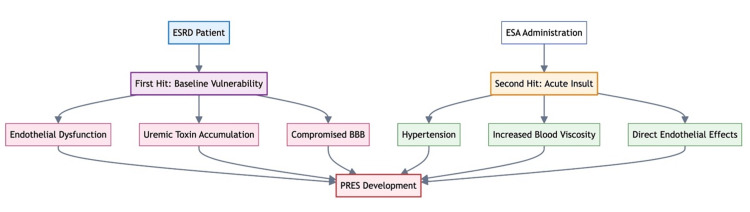
Two-hit hypothesis for the development of PRES in patients receiving EPO analogs. Image credit: Avinash K. Jha. ESRD, end-stage renal disease; BBB, blood-brain barrier; ESA, erythropoietin-stimulating analog/EPO analog; PRES, posterior reversible encephalopathy syndrome

The hypertension caused by these drugs can develop between two weeks and four months after starting therapy [5]. PRES is a relatively rare side effect of EPO, although occasional case reports have highlighted this association [5-7]. Applying the Naranjo and WHO-UMC (Health Organization-Uppsala Monitoring Center) criteria yielded a score of 5, classifying the association between EPO analogs and PRES as *probable* for epoetin alfa, while darbepoetin alfa remained *possible *because of the absence of indexed cases. The clinical course in this patient's first episode of PRES after darbepoetin and recurrence with rechallenge provides strong circumstantial evidence for causality. We propose a two-hit hypothesis for the development of PRES with EPO analog use, as illustrated in Figure 5.

Management of PRES often requires an ICU setting. In one study, 70% of patients with PRES needed ICU care for PRES-related complications, including status epilepticus [8]. Therefore, critical care teams should be vigilant, especially in patients with renal disease who are treated with EPO analogs. Early MRI is crucial for diagnosis. Brain MRI shows bilateral cortico-subcortical vasogenic edema, which manifests in three anatomical patterns seen in approximately 70% of patients: a dominant parieto-occipital pattern (22%), a holohemispheric watershed pattern (23%), and a superior frontal sulcus pattern (27%) [3,9]. Regarding clinical features, seizures are common in PRES, occurring in up to 81% of patients [10], and most often present as generalized tonic-clonic episodes [11], with a tendency to recur. In a retrospective review of 49 patients with PRES, 17.6% experienced recurrent generalized tonic-clonic seizures [12].

Management involves controlling BP and stopping the offending agent. In specific situations, weaning or switching to subcutaneous dosing with a lower target hemoglobin level may be safer [5,6].

This case highlights the significance of regular medication reconciliation and interdisciplinary communication in patients with complex medication regimens.

## Conclusions

EPO analog-induced PRES is a rare but potentially reversible cause of altered mental status in patients with ESRD in the ICU. Early detection, discontinuation of the offending agent, and provision of supportive care can lead to a full recovery. Increased clinical suspicion is crucial for timely diagnosis and effective intervention. Key points are that PRES may occur secondary to hypertension caused by an EPO analog, especially in patients with ESRD. Stopping EPO analogs and carefully managing blood pressure are vital components of treatment. Re-administering EPO analogs could cause recurrence; therefore, considering an alternative dosing regimen is advisable.
